# CdIn_2_S_4_/In(OH)_3_/NiCr-LDH Multi-Interface Heterostructure Photocatalyst for Enhanced Photocatalytic H_2_ Evolution and Cr(VI) Reduction

**DOI:** 10.3390/nano11113122

**Published:** 2021-11-19

**Authors:** Rao Fu, Yinyan Gong, Can Li, Lengyuan Niu, Xinjuan Liu

**Affiliations:** 1 College of Materials and Chemistry, China Jiliang University, Hangzhou 310018, China; furao0802@163.com; 2 Institute of Optoelectronic Materials and Devices, College of Optical and Electronic Technology, China Jiliang University, Hangzhou 310018, China; canli1983@gmail.com (C.L.); niulengyuan@163.com (L.N.); lxj669635@126.com (X.L.)

**Keywords:** photocatalysis, nano-heterostructures, charge separation, LDHs, CdIn_2_S_4_, In(OH)_3_

## Abstract

The development of highly active and stable photocatalysts, an effective way to remediate environment pollution and alleviate energy shortages, remains a challenging issue. In this work, a CdIn_2_S_4_/In(OH)_3_ nanocomposite was deposited in-situ on NiCr-LDH nanosheets by a simple hydrothermal method, and the obtained CdIn_2_S_4_/In(OH)_3_/NiCr-LDH heterostructure photocatalysts with multiple intimate-contact interfaces exhibited better photocatalytic activity. The photocatalytic H_2_ evolution rate of CdIn_2_S_4_/In(OH)_3_/NiCr-LDH increased to 10.9 and 58.7 times that of the counterparts CdIn_2_S_4_ and NiCr-LDH, respectively. Moreover, the photocatalytic removal efficiency of Cr(VI) increased from 6% for NiCr-LDH and 75% for CdIn_2_S_4_ to 97% for CdIn_2_S_4_/In(OH)_3_/NiCr-LDH. The enhanced photocatalytic performance was attributed to the formation of multi-interfaces with strong interfacial interactions and staggered band alignments, which offered multiple pathways for carrier migration, thus promoting the separation efficiency of photo-excited electrons and holes. This study demonstrates a facile method to fabricate inexpensive and efficient heterostructure photocatalysts for solving environmental problems.

## 1. Introduction

Against the background of the fossil energy shortage and water pollution by heavy metals and organic contaminants, it is essential to develop new technologies for environmental remediation and the production of green and renewable energy. Photocatalysis has drawn significant interest because of its ability to produce chemical energy, decompose organic pollutants, and detoxify heavy metal contamination by utilizing solar energy [1,2,3,4,5]. The performance of this environmentally-friendly technique mainly relies on the ability of photo-excited electrons (e^−^) and holes (h^+^) in a semiconductor photocatalyst to migrate to the surface and participate in a series of photocatalytic redox reactions. For instance, photo-generated electrons in a semiconductor with negative enough conduction band (CB) potential can react with H_2_O to produce H_2_, a promising substitute for traditional fossil fuels [1,2,6]. Hexavalent chromium ions (Cr(VI)), a serious threat to human health due to their solubility, acute toxicity and high carcinogenicity, can also be reduced to the nontoxic Cr(III) by photo-excited electrons [7,8]. 

To develop highly efficient and cost-effective photocatalysts, various semiconductors have been investigated during the past few decades, including metal oxides, metal sulfides, (oxy)nitrides, etc. [9,10,11]. Unfortunately, no satisfactory semiconductor photocatalyst has so far been obtained for practical applications. One of the bottleneck problems restricting the performance of single component semiconductor photocatalyst is the swift recombination of photo-generated e^−^ and h^+^ pairs. Fabrication of heterostructure photocatalysts is a promising strategy since this method makes it feasible to spatially separate photo-excited electrons and holes, and thus achieve higher photocatalytic efficiency than the component semiconductors [12,13,14,15,16,17,18,19,20]. For example, Jiang and coworkers fabricated CsPbBr_3_/Bi_2_WO_3_ heterostructure photocatalysts by a simple electronic self-assembly process, and successfully improved the CO_2_ photoreduction activity to more than 12-fold that of pristine CsPbBr_3_ [21]. Han et al. constructed ZnIn_2_S_4_/BiVO_4_ photocatalysts by assembling ZnIn_2_S_4_ nanosheets on BiVO_4_ decahedrons, and increased the photocatalytic CO yield by 9 times in comparison with pristine ZnIn_2_S_4_ [22]. 

Layered double hydroxides (LDHs) have garnered increasing attention in the field of photocatalysis because of their characteristic two-dimensional (2D) layered structure, stable photochemical properties and tunable electronic properties [23,24,25,26]. The general formula of LDHs can be expressed as [M1−x2+Mx3+(OH)2]x+(An−)xn·mH2O, where M^2+^ and M^3+^ denote divalent (Mg^2+^, Co^2+^, Ni^2+^, Cu^2+^, Zn^2+^, etc.) and trivalent (Al^3+^, Cr^3+^, Fe^3+^, Co^3+^, etc.) metal cations, respectively, and A^n−^ represents interlayer anions (CO_3_^2−^, NO^3−^, Cl^−^, CH_3_COO^−^, etc.) [23,26,27]. For a typical LDH crystal, M^2+^, M^3+^ and OH^-^ groups constitute positively charged main laminas, ${\left[M_{1-x}^{2+}M_{x}^{3+}{\left(\mathrm{OH}\right)}_{2}\right]}^{x+}$, which are stacked vertically. Meanwhile, charge-balancing anions A^n−^ and water molecules are sandwiched between these positively charged 2D hydroxide layers. The band gap energy (E_g_) and conduction band/valence band potentials can be modulated by varying M^2+^ and M^3+^, and the charge density of 2D hydroxide layers depends on the atomic content of M^3+^ (0.2 ≤ x ≤ 0.33). LDHs seem to be a good candidate for the development of heterostructure photocatalytic systems, and various LDH-involved composite photocatalysts with enhanced performance have been reported, such as MoS_2_/CoAl-LDH [28], CdS/NiCo-LDH [29], MoS_2_/NiFe-LDH [30], Cu_2_O/NiFe-LDH [31], and g-C_3_N_4_/ZnAl-LDH [32].

Owing to their strong visible-light absorption ability and negative enough CB potential [33], metal sulfides (e.g., CdIn_2_S_4_, ZnIn_2_S_4_, and CuInS_2_) have attracted extensive interests in various photocatalytic applications, including photocatalytic conversion of CO_2_ to hydrocarbon fuels [34], hydrogen production [35,36], Cr(VI) photoreduction [37], and organics degradation [37,38]. However, the photocatalytic performance of single component metal sulfide materials is still not satisfactory, a main reason being the sluggish separation and transfer kinetics of charge carriers. It has been proposed that the construction of heterostructure photocatalysts by coupling ternary metal sulfides with suitable semiconductors is a good solution to suppress the swift recombination of photo-generated e^-^ and h^+^ pairs. Different metal sulfide-involved photocatalytic systems with enhanced photocatalytic activity have been reported, including WO_3_/CdIn_2_S_4_ [39], In_2_S_3_/CdIn_2_S_4_ [40], WO_2.7_/ZnIn_2_S_4_ [41], CuInS_2_/Bi_2_MoO_6_ [42], and Co_8_S_7_/ZnIn_2_S_4_ [43]. As indium ions can hydrolyze into In(OH)_3_ by adjusting hydrothermal growth conditions (e.g., pH value, concentration of released S^2−^), it is convenient to synthesize ternary metal sulfides/In(OH)_3_ nanocomposite photocatalysts by a simple one-step hydrothermal method. In such a system, it is expected that the less negative CB potential of In(OH)_3_, and the possibility of shared In ions (multiple interfaces with intimate contact), would facilitate spatial separation of charge carriers and thus improve photocatalytic performance. As expected, such nanocomposites with enhanced photocatalytic activity have been reported, including ZnIn_2_S_4_/In(OH)_3_ [44,45,46], ZnIn_2_S_4_/In(OH)_3_/NiS [47], ZnIn_2_S_4_/In(OH)_3_/ZnWO_4_ [48], and CdIn_2_S_4_/In(OH)_3_/Zn_2_GeO_4_ [49].

Inspired by the above benefits, we attempt in this work to construct a CdIn_2_S_4_/In(OH)_3_/NiCr-LDH ternary heterostructure photocatalyst. Considering the fact that CdIn_2_S_4_ is a semiconductor photocatalyst with strong visible-light absorption (E_g_ = 2.20 eV) and CB potential more negative than H^+^/H_2_ potential [50], and NiCr-LDH also possesses strong visible-light absorption due to the uniform distribution of multivalent Cr ions within the 2D hydroxide layers and high oxidation potential [51], it seems to be a reasonable route to develop heterostructure photocatalysts involving CdIn_2_S_4_ and NiCr-LDH. Moreover, the abundant hydroxyl groups on the surface of NiCr-LDH make it convenient to in-situ growth of In(OH)_3_, and thus it is feasible to deposit CdIn_2_S_4_ and In(OH)_3_ nanocomposite on NiCr-LDH by one-step hydrothermal growth. The as-prepared multi-component photocatalytic systems are demonstrated to be effective and recyclable for photocatalytic H_2_ production and Cr(VI) reduction. The enhanced photocatalytic performance of CdIn_2_S_4_/In(OH)_3_/NiCr-LDH are attributed to boosted separation and migration of charge carriers due to staggered band alignment, and multiple intimate-contact interfaces. This work demonstrates a simple strategy to construct LDH-based ternary photocatalysts, which generally involve two or more complicated synthesis procedures and are relatively time-consuming. The results of this work could be beneficial for further construction of other multi-component photocatalytic systems for environmental and energy applications. Moreover, NiCr-LDH provides a good platform for the fabrication of heterostructure photocatalysts. To date, there are very limited research reports on NiCr-LDH-based composites in the field of photocatalysis.

## 2. Materials and Methods

### 2.1. Synthesis of NiCr-LDH

Ni(NO_3_)_2_ 6H_2_O (1.8 mmol), Cr(NO_3_)_3_ 9H_2_O (0.6 mmol) and urea (8 mmol) were dissolved in 60 mL deionized (DI) water, which was then transferred into a 100 mL Teflon-lined stainless steel autoclave and heated at T = 120, 150, 180 and 190 °C for 10 h. After being naturally cooled, the precipitates were collected by centrifugation, thorough washed with DI water, and drying at 60 °C in vacuum overnight. The obtained NiCr-LDH samples were labeled as NC-T, where T stands for the temperature of hydrothermal growth.

### 2.2. Synthesis of CdIn_2_S_4_/In(OH)_3_/NiCr-LDH

As-prepared NC-T was dispersed in 60 mL H_2_O by ultrasonication treatment (1.667 g/L). Stoichiometric amounts of Cd(NO_3_)_2_·4H_2_O (0.2 mmol), In(NO_3_)_3_·4.5H_2_O (0.4 mmol) and thioacetamide (TAA, 0.8 mmol) were dissolved in the suspension under magnetic stirring. The mixture was then transferred into a 100 mL Teflon-lined stainless steel autoclave and reacted at 180 °C for 12 h. After being naturally cooled, the products were centrifuged, washed thoroughly by DI water, and dried at 60 °C in vacuum overnight. Such obtained CdIn_2_S_4_/In(OH)_3_/NiCr-LDH heterostructure photocatalysts were referred to as HP-T (T is the same as that of used NC-T). The obtained HP-180 contains ~12 wt%, 21 wt% and 67 wt% of CdIn_2_S_4_, In(OH)_3_, NiCr-LDH, respectively. We also prepared CdIn_2_S_4_/In(OH)_3_/NiCr-LDH with excess amounts of TAA under similar conditions as HP-180. HP-180-1 and HP-180-2 correspond to the samples prepared using 1.2 mmol and 1.6 mmol of TAA (molar ratio of TAA/Cd(NO_3_)_2_·4H_2_O equal to 6 and 8), respectively. HP-180-3 and HP-180-4 were synthesized by changing the amounts of NiCr-LDH to 0.5 times (50 mg) and 3 times (300 mg) that used for HP-180, respectively. 

As a comparison, CdIn_2_S_4_ was prepared under the same conditions without the incorporation of NiCr-LDH, and labeled as CIS-4, CIS-4.4, CIS-6 and CIS-8 (“4, 4.4, 6, and 8” corresponds to the molar ratio of TAA/Cd(NO_3_)_2_·4H_2_O). Cd(NO_3_)_2_·4H_2_O, In(NO_3_)_3_·4.5H_2_O and C_2_H_5_NS were purchased from Aladdin, Shanghai, China. Ni(NO_3_)_2_·6H_2_O and ethonal were bought from Sinopharm Chemical Reagent Co. Ltd, Shanghai, China. Cr(NO_3_)_3_ 9H_2_O was bought from Macklin, Shanghai, China.

### 2.3. Evaluation of Photocatalytic Performance

Photocatalytic H_2_ production. The experiments were carried out on a Lab Solar system (Labsolar-III AG, Perfectlight Technology, Co. Ltd., Beijing, China). Briefly, 50 mg as-prepared photocatalysts were dispersed in 80 mL solution of Na_2_S (0.35 M) and Na_2_SO_3_ (0.25 M) in a 300 mL quartz reactor. The suspension was vacuumed and stirred in the dark for 30 min, and then exposed to light irradiation emitted from a 300 W Xenon lamp (PLS-SXE 300, PerfectLight Technology, Co. Ltd., Beijing, China) positioned above the reactor (Figure S1). The photocatalytic generated H_2_ from water splitting was quantitatively analyzed online every hour by a gas chromatography (GC2014, TCD, 5 Å molecular sieve, Shimadzu, Japan). The apparent quantum yield (AQY) was measured under similar conditions, except that a 420 nm bandpass filter (FWHM: 21 nm, DT420, PerfectLight Technology, Beijing, China) was installed on the Xe lamp. The light intensity was 5.01 mW cm^−2^ and the amount of evolved H_2_ was 14.93 μmol for 4 h. The AQY was calculated based on the following equation (detailed calculation was given in SI):

$$\mathrm{AQY}\;\left(\%\right)=\frac{\mathrm{number}\;\mathrm{of}\;\mathrm{reacted}\;\mathrm{electrons}}{\mathrm{Number}\;\mathrm{of}\;\mathrm{incident}\;\mathrm{photons}}\times 100\%\;\mathrm{AQY}\;\left(\%\right)=\frac{2\times {\mathrm{number}\;\mathrm{of}\;\mathrm{evolved}\;H}_{2}\;\mathrm{molecules}}{\mathrm{number}\;\mathrm{of}\;\mathrm{incident}\;\mathrm{photons}}\times 100\%$$

$$\mathrm{AQY}\;\left(\%\right)=\frac{2\times {\mathrm{number}\;\mathrm{of}\;\mathrm{evolved}\;H}_{2}\;\mathrm{molecules}}{\mathrm{number}\;\mathrm{of}\;\mathrm{incident}\;\mathrm{photons}}\times 100\%$$

Cr(VI) photoreduction. The experiments were carried out on a multi-tube photocatalytic reaction system (JOYN-GHX-AC, Qiaoyue Electronics, Shanghai, China). A total of 30 mg as-prepared photocatalysts were dispersed in Cr(VI) solution (50 mL, 50 ppm) in 80 mL quartz tubes, and stirred in dark for 30 min. Then, a 400 W Mercury light bulb equipped with cutoff filters (λ ≥ 400 nm) was turned on to shine light on the suspension. At fixed time intervals, 4 mL of suspension was sampled and centrifuged to remove the residual catalysts. Finally, the Cr(VI) content was monitored by comparing the change in absorbance on a Shimadzu UV-V is 3600 spectrometer (Shimadzu, Japan). To test the stability, HP-180 was washed after each run and Cr(VI) solution was refilled.

## 3. Results and Discussion

### 3.1. Characterization of Photocatalysts

**Figure 1:**
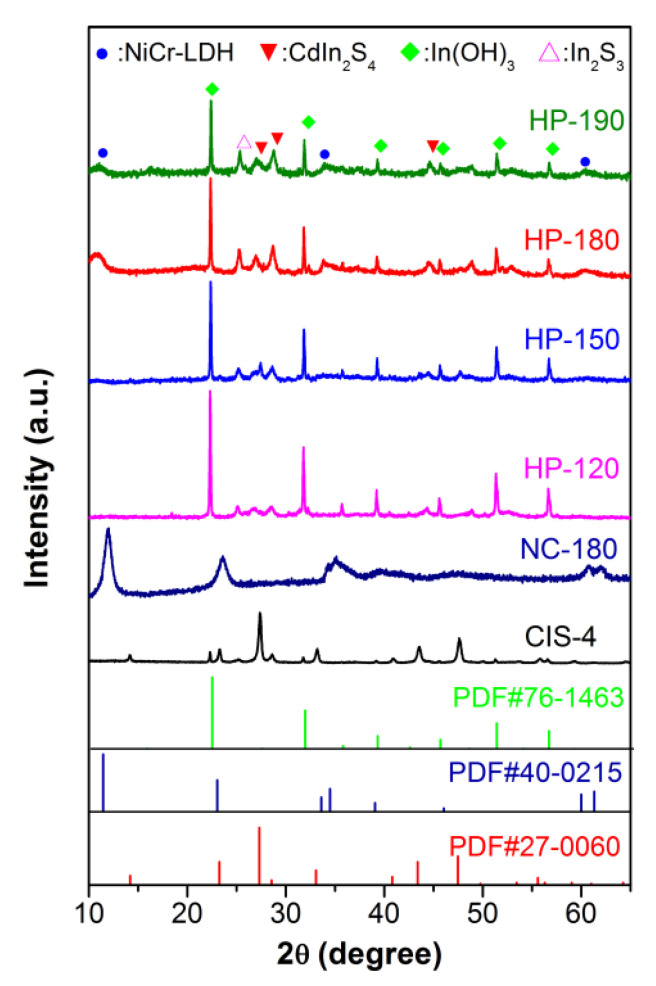
XRD patterns of HP-120, HP-150, HP-180 and HP-190 together with NC-180 and CIS-4.

The structure and morphology of as-prepared photocatalysts were investigated by XRD, SEM, and TEM analysis. Figure 1 showed the recorded XRD curves of the CdIn_2_S_4_/In(OH)_3_/NiCr-LDH composite photocatalysts HP-120, HP-150, HP-180 and HP-190 together with NC-180 and CIS-4. Figure S2a displayed XRD patterns of NC-120, NC-150, NC-180 and NC-190. It can be found that pure NiCr-LDH showed diffraction peaks at 2θ = 11.6°, 23.1°, 33.8°, 34.6°, 39.1°, 46.2°, 60.1°, and 61.5°, which can be assigned to (003), (006), (101), (012), (015), (018), (110) and (113) planes of LDHs with CO_3_^2−^ as interlayer anions (JCPDS No. 40-0215). The weak and broad diffraction peaks of NC-120 implied low crystalline quality which might be caused by the relatively low growth temperature. Figure S2b showed the XRD curves of CIS-4 and CdIn_2_S_4_ prepared at higher concentrations of TAA (molar ratio of TAA/Cd(NO_3_)_2_·4H_2_O equal to 4.4, 6 and 8, CIS-4.4 CIS-6, and CIS-8, respectively). The dominant XRD pattern of CIS-4 can be assigned to the cubic phase of CdIn_2_S_4_ (JCPDS No. 27-0060), and the diffraction peaks at 2θ = 14.2°, 23.2°, 27.3°, 28.5°, 33.1°, 40.8°, 43.4°, 47.5° and 55.6° correspond to (111), (220), (311), (222), (400), (422), (511), (440), and (533) reflections, respectively. In addition, weak diffraction signals observed at 2θ = 22.3°, 31.7°, 39.1°, 45.5°, 51.3°, and 56.6° are associated with (200), (220), (222), (400), (420) and (422) reflections from cubic phase of In(OH)_3_ (JCPDS No. 76-1463). We note that the diffraction signal of In(OH)_3_ is much weaker than that of CdIn_2_S_4_, and the intensity ratio of the dominant peaks is $\frac{I_{\left(311\right)}^{CdIn_{2}S_{4}}}{I_{\left(200\right)}^{In{\left(OH\right)}_{3}}}=4$, implying In(OH)_3_ is impurity phase with low content in CIS-4. No diffraction peaks of In(OH)_3_ phase were detected in CIS-4.4 CIS-6, and CIS-8. The weak peak at 2θ = 25.3° is related to tetragonal In_2_S_3_ (JCPDS No. 73-1366). As shown in Figure 1, HP-120, HP-150, HP-180 and HP-190 have similar XRD patterns, which are composed of diffraction signals from both NiCr-LDH and CIS-4, suggesting successful preparation of CdIn_2_S_4_/In(OH)_3_/NiCr-LDH composite photocatalysts. Compared to CIS-4, the diffraction peaks associated with In(OH)_3_ became stronger in HP-180 and the intensity ratio decreased to $\frac{I_{\left(311\right)}^{CdIn_{2}S_{4}}}{I_{\left(200\right)}^{In{\left(OH\right)}_{3}}}=0.3$, implying increased content of In(OH)_3_. A plausible explanation might be that the abundant hydroxyl groups on the surface of NiCr-LDH can bond with In^3+^ ions during the hydrothermal growth and thus promote the formation of In(OH)_3_. Figure S2c showed the XRD curves of CdIn_2_S_4_/In(OH)_3_/NiCr-LDH prepared with increasing amounts of TAA. Compared to HP-180, it can be found that the characteristic peak of In(OH)_3_ becomes weaker in HP-180-1, and disappears in HP-180-2, indicating that the amounts of TAA could affect the relative content of CdIn_2_S_4_ and In(OH)_3_. Figure S2d displayed the XRD patterns of CdIn_2_S_4_/In(OH)_3_/NiCr-LDH synthesized by using different amounts of NiCr-LDH. HP-180 and HP-180-4 exhibited similar diffraction patterns. For HP-180-3, the (003) diffraction peak of NiCr-LDH can hardly be observed, which might be due to reduced amount of NiCr-LDH (0.5 times that of HP-180). In addition, characteristic peaks associated with CdIn_2_S_4_ became more pronounced compared to HP-180. 

**Figure 2:**
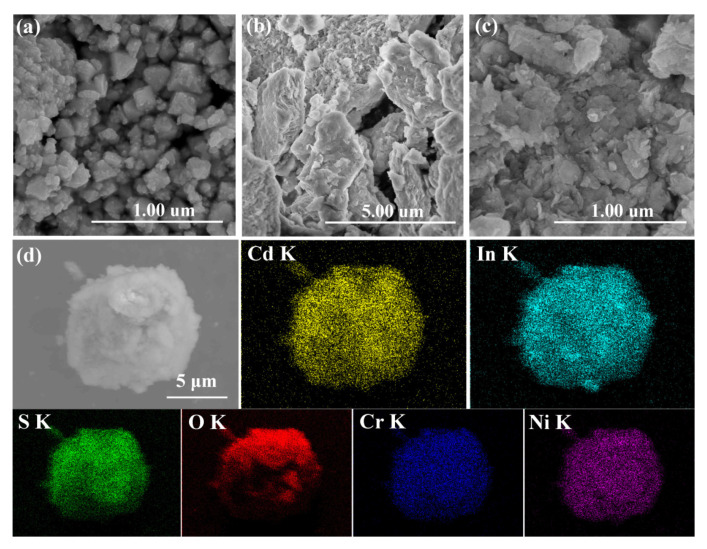
(**a**–**c**) SEM images of CIS-4, NC-180 and HP-180, (**d**) EDX mapping of Cd, In, S, O, Cr, and Ni elements in HP-180.

**Figure 3:**
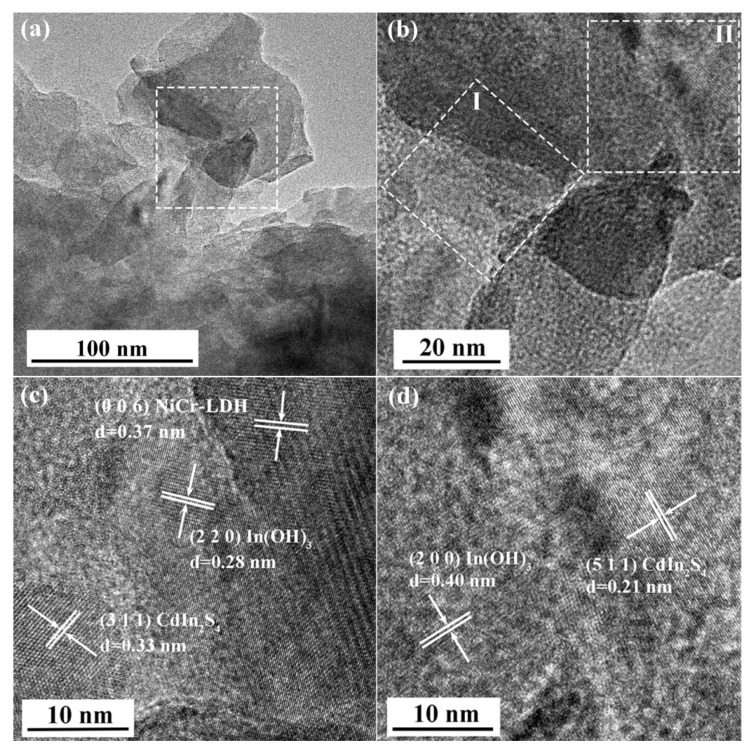
(**a**,**b**) TEM and (**c**,**d**) HRTEM images of HP-180, where c and d correspond to regions I and II, respectively.

Figure 2a–c display SEM images of CIS-4, NC-180 and HP-180, respectively. CIS-4 exhibited an octahedral morphology with small flakes and particles on the surface, while pristine NiCr-LDH showed an irregular shape with an aggregated sheet-like structure. In comparison, the HP-180 photocatalytic system revealed an interwoven-sheet structure composed of nanoflakes. Figure 2d showed SEM EDX mapping. It revealed that Cd, In, S, Cr and Ni elements are homogeneously distributed in HP-180. O is detected in the whole analyzed area but its distribution is less even. This result was confirmed by TEM observation. In the HRTEM image in Figure 3c and Figure S3b, the lattice spacing of 0.33, 0.28 and 0.37 nm correspond to (311), (220) and (006) crystal planes of CdIn_2_S_4_, In(OH)_3_ and NiCr-LDH, respectively. In Figure 3d, the lattice spacing of 0.21 and 0.40 nm correspond to (511) and (200) crystal planes of CdIn_2_S_4_ and In(OH)_3_, respectively. In Figure S3b, the lattice spacing 0.26 nm corresponds to (012) crystal plane of NiCr-LDH. The HRTEM observation suggested the formation of a composite photocatalyst with multiple intimate-contact interfaces, which could facilitate the transfer and separation of charge carriers across the heterojunction.

**Figure 4:**
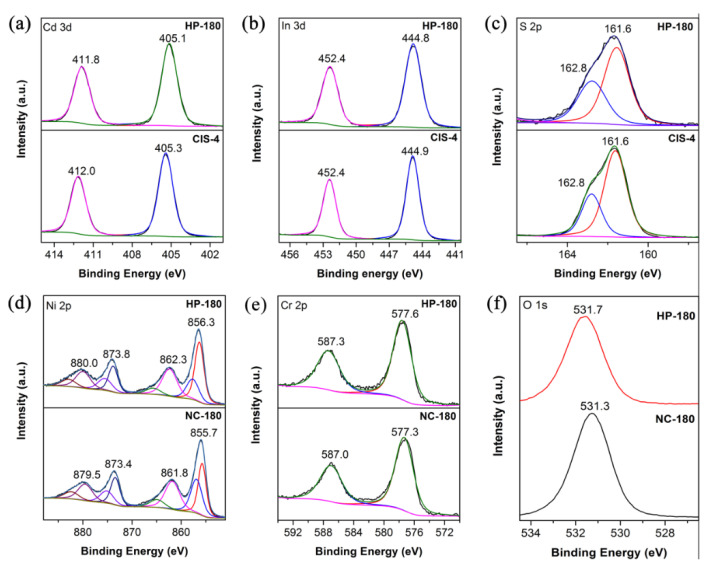
High-resolution XPS spectra of CIS-4, NC-180 and HP-180 (**a**) Cd 3d, (**b**) In 3d, (**c**) S 2p, (**d**) Ni 2p, (**e**) Cr 2p, (**f**) O 1s spectra.

XPS measurements were carried out to analyze the surface elemental composition and chemical states of the as-prepared photocatalysts. As shown in the survey spectra (Figure S4), CIS-4 was composed of Cd, In, and S, while NC-180 was composed of Ni, Cr, C and O elements. For HP-180, the measured XPS spectrum showed the signals of Cd, In, S, Ni, Cr, C, and O elements, suggesting the hybridization of CIS-4 and NC-180. Figure 4 showed the high-resolution spectra of Cd, In, S, Ni, Cr and O. The Cd 3d spectrum of CIS-4 exhibited two main peaks at 405.3 and 412.0 eV, which are associated with Cd 3d_5/2_ and Cd 3d_3/2_ of Cd^2+^, respectively [50,52]. After coupling with NiCr-LDH, the binding energies of Cd 3d_5/2_ and Cd 3d_3/2_ of HP-180 were decreased to 405.1 and 411.8 eV, respectively. The In 3d spectrum of CIS-4 had two peaks with binding energies of 444.9 and 452.4 eV, corresponding to In 3d_3/2_ and 3d_5/2_ of In^3+^, respectively [41,52]. For HP-180, the In 3d_5/2_ peak was negatively shifted to 444.8 eV. As shown in Figure 4c, the 161.6 and 162.8 eV peaks could be assigned to S 2p_3/2_ and S 2p_1/2_, respectively [40]. No obvious shift was observed in the S 2p spectrum of HP-180. The Ni 2p signal of pure NiCr-LDH can be deconvoluted into doublet peaks centered at 855.7 and 873.4 eV associated with Ni 2p_3/2_ and Ni 2p_1/2_ of Ni^2+^ oxidation state, and peaks at 861.8 and 879.5eV originated from Ni^3+^ state [53,54], indicating the co-existence of Ni^2+^ and N^3+^ ions. In the Ni 2p XPS spectrum of HP-180, the binding energy peaks were positively shifted to 856.3, 862.3, 873.8 and 880.0 eV, respectively. The Cr 2p spectrum of pure NiCr-LDH revealed two characteristic peaks at 577.3 and 587.0 eV, which can be attributed to Cr^3+^ in LDH lattice [55], while Cr 2p peaks of HP-180 were positively shifted to 577.6 and 587.3 eV, respectively. The binding energy of O 1s was increased from 531.3 eV for NiCr-LDH to 531.7 eV for HP-180. The decreased binding energies of Cd 3d, and In 3d, and increased binding energies of Ni 2p, Cr 2p and O 1s in CdIn_2_S_4_/In(OH)_3_/NiCr-LDH indicated that there was intimate interfacial contact and strong chemical interaction among CdIn_2_S_4_, In(OH)_3_ and NiCr-LDH, which may suppress electron-hole recombination. Combining the characterization results of XRD, SEM, TEM, and XPS analysis, CdIn_2_S_4_/In(OH)_3_/NiCr-LDH heterostructure photocatalysts with strong interfacial interaction were obtained, which could promote spatial charge separation and lead to improved photocatalytic performance.

**Figure 5:**
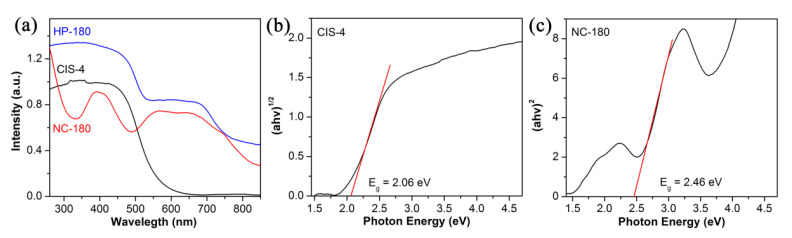
(**a**) UV-Vis absorption spectra of CIS-4, NC-180 and HP-180; (**b**,**c**) Tauc-plots of CIS-4 and NC-180, respectively.

Since the solar light response regime also plays an important role in photocatalytic efficiency, UV-Vis diffuse reflectance spectra were measured to investigate the absorption ability of the as-prepared photocatalysts. As shown in Figure 5a, CIS-4 exhibited strong absorption until the wavelength reached around 570 nm. NiCr-LDH could respond to a wider solar spectrum, with two broad absorption bands in the visible region between 330–480 nm and 490–850 nm, which are associated with the d–d transitions of metal ions distributed in the 2D hydroxide layers of LDH [51,56]. The CdIn_2_S_4_/In(OH)_3_/NiCr-LDH heterostructure photocatalyst not only had strong light absorption up to 530 nm, similar to that of CdIn_2_S_4_, but also exhibited an absorption band in the region of 530–850 nm, which was consistent with NiCr-LDH. The enhanced optical absorption would help to utilize solar energy and generate more photo-generated charges, thereby enhancing the photocatalytic activity. As shown in Figure 5b,c, the band gap energies (E_g_) of CdIn_2_S_4_ and NiCr-LDH were extracted via $\alpha h\nu =A{\left(h\nu -E_{g}\right)}^{n/2}$ and equal to 2.06 and 2.46 eV, respectively. It was noted that n = 4 (indirect band gap) and n = 1 (direct band gap) were used for CdIn_2_S_4_ and NiCr-LDH, respectively. Figure S5a,b showed the band structures of CdIn_2_S_4_ and NiCr-LDH from band density functional theory (DFT) calculations. For CdIn_2_S_4_, VB maximum (VBM) and CB minimum (CBM) were located at different point of the Brillouin zone, suggesting the compound is an indirect band gap semiconductor for NiCr-LDH. The VBM and CBM are positioned at the same point of the Brillouin zone, implying it is a direct band gap. The calculated energy gaps are 2.11 and 1.89 eV for CdIn_2_S_4_ and NiCr-LDH, respectively.

### 3.2. Photocatalytic Activity

**Figure 6:**
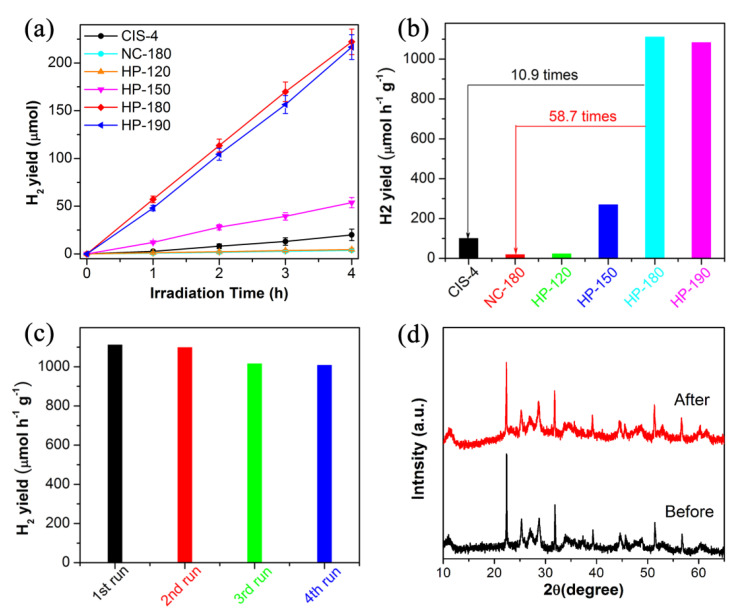
Comparison of photocatalytic activity (**a**) H_2_ production versus time and (**b**) H_2_ evolution rate over pristine CIS-4, NC-180, HP-120, HP-150, HP-180 and HP-190; (**c**) recyclability experiment of photocatalytic H_2_ generation over HP-180; (**d**) XRD patterns of HP-180 before and after test.

The photocatalytic H_2_ production activities from water splitting over as-prepared photocatalysts were measured under a 300 W Xe lamp irradiation. Figure 6 showed the results of HP-120, HP-150, HP-180 and HP-190, which were prepared by using the same amounts of Cd(NO_3_)_2_·4H_2_O, In(NO_3_)_3_·4.5H_2_O, thioacetamide and NiCr-LDH, except the growth temperature of NiCr-LDH was increased from 120 to 190 °C. For comparison, H_2_ evolution of the sample prepared under similar conditions as HP-180 without the introduction of NiCr-LDH (CIS-4) and pure NiCr-LDH (NC-180) were also plotted. As expected, the heterostructure photocatalysts HP-150, HP-180 and HP-190 exhibited better photocatalytic H_2_ production performance. In particular, HP-180 presented the highest H_2_ evolution rate of 1093 μmol·g−1·h−1, which is 10.9 and 58.7 times the counterparts of CIS-4 and NC-180, respectively. For HP-180, the calculated AQY is 1.7% at 420 nm. Table S1 lists the photocatalytic H_2_ evolution of LDH-based heterostructures. It can be found that the AQY of HP-180 is slightly higher than the reported value for ZnS/ZnIn-LDH (AQY=1.3% at 415 nm) [57]. Although the AQY of HP-180 is lower than the reported values for g-C_3_N_4_@pDA/NiCo-LDH (4.5% at 420 nm) [58] and phosphorylated-NiAl-LDH/g-C_3_N_4_ (4.7% at 420 nm) [59], they involved three steps of growth. The simple strategy to obtain ternary heterostructure photocatalysts in this work still has its advantage and could shed light on further design, and easy and cost-effective preparation of new LDH-based multicomponent nanocomposite photocatalysts. It was noted that the weak catalytic activity of HP-120 might be due to the poor crystalline quality of NiCr-LDH grown at 120 °C. To examine the long-term reusable life and stability, HP-180 was reused four consecutive times (Figure 6c). There was no obvious decrease in photocatalytic activity of HP-180, and the H_2_ evolution rate remained at 91% that of freshly prepared sample after four consecutive runs. Moreover, there were no observable changes in the XRD pattern of the sample collected after the cycling test in comparison with that of as-prepared sample. We note that we also investigated the influence of the amount of catalysts on H_2_ evolution. As shown in Figure S6, 50 mg of HP-180 leads to higher photocatalytic H_2_ production. In this work, all H_2_ production measurements were conducted using 50 mg photocatalysts.

Figure S7a,b showed H_2_ production activities of HP-180, HP-180-1 and HP-180-2 with decreased relative content of In(OH)_3_/CdIn_2_S_4_, which was estimated to be around 2.3, 0.45 and 0, respectively, due to increased amounts of TAA used during synthesis. It can be found that the amount of evolved H_2_ decreased in the following sequence: HP-180 > HP-180-1 > HP-180-2. This indicates that the relative amount of CdIn_2_S_4_ to In(OH)_3_ could affect the photocatalytic activity. Moreover, the stronger H_2_ evolution activity of HP-180 compared to HP-180-2 implies that the construction of ternary CdIn_2_S_4_/In(OH)_3_/NiCr-LDH is helpful to further improve photocatalytic H_2_ evolution compared to CdIn_2_S_4_/NiCr-LDH. Additionally, Figure S7c,d compared the H_2_ generation over samples prepared by using 50, 100 and 300 mg of NiCr-LDH, corresponding to HP-180-3, HP-180 and HP-180-4, respectively. The H_2_ evolution rate of HP-180 is 9.11 and 7.34 times that of HP-180-3 and HP-180-4. 

**Figure 7:**
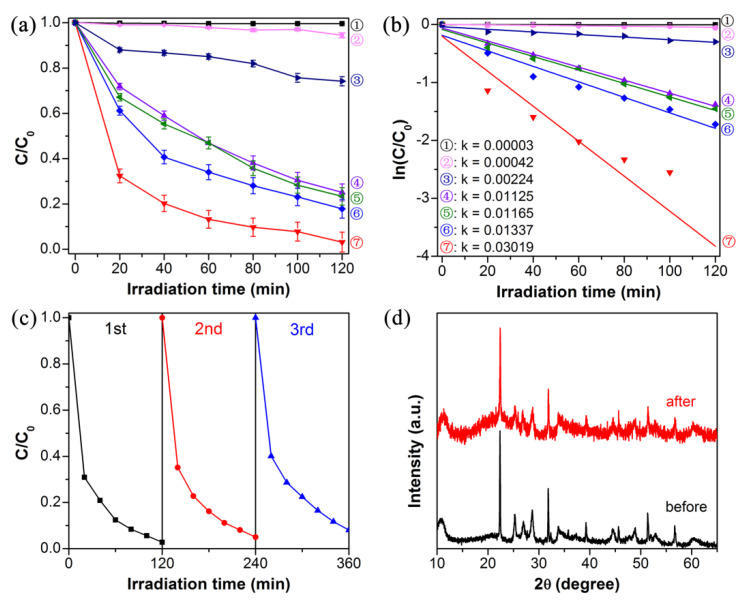
(**a**) Photocatalytic reduction efficiency and (**b**) pseudo first-order kinetic fitting curves of aqueous Cr(VI) over ① no catalyst, ② NC-180, ③ mixture of CdIn_2_S_4_, In(OH)_3_ and NiCr-LDH, ④ CIS-4, ⑤ HP-180-2, ⑥ HP-180-1, and ⑦ HP-180. (**c**) recyclability experiment of Cr(VI) photoreduction over HP-180; and (**d**) XRD curves of HP-180 before and after stability test.

In addition, the prepared photocatalysts were also tested for photocatalytic Cr(VI) reduction. Figure 7a,b showed the photocatalytic reduction efficiency of Cr(VI) over NC-180, CIS-4, HP-180, HP-180-1, HP-180-2 and the mixture of CdIn_2_S_4_, In(OH)_3_ and NiCr-LDH. Without the presence of catalysts, there was barely any change in Cr(VI) concentration. The presence of CIS-4, HP-180, HP-180-1 and HP-180-2 photocatalysts resulted in an obvious decrease in Cr(VI) concentration, suggesting that they play an important role in the photocatalytic reduction of Cr(VI). Moreover, the Cr(VI) removal efficiency decreased in the following sequence: HP-180 > HP-180-1 > HP-180-2 > CIS-4 > mixture > NC-180. Among all the tested photocatalysts, HP-180 exhibited the highest photoreduction efficiency at 97% after 120 min illumination, which is higher than CIS-4 at 75% and NC-180 at 6%. The kinetic rate constant of HP-180 is 0.03019 min^−1^, which is 1.7 and 74.5 times higher than those of CIS-4 (0.01125 min^−1^) and NC-180 (0.0004 min^−1^). The scavenger tests revealed that electrons played a dominant role in the photocatalytic reduction of Cr(VI) in this work (Figure S8). Figure 7c displayed the results of stability tests of Cr(VI) reduction over HP-180. It can be found that the removal efficiency remains at 94% that of the as-prepared sample after three consecutive runs, and the slight decrease in efficiency might be caused by loss of catalyst due to washing after each run. Furthermore, there were no observable changes in the XRD pattern of the sample collected after the cycling test. Consequently, the results of the photocatalytic experiments suggested that CdIn_2_S_4_/In(OH)_3_/NiCr-LDH heterostructure photocatalysts are capable of effective H_2_ evolution from water splitting and reduction of Cr(VI) with good stability.

### 3.3. Plausible Photocatalytic Mechanism

**Figure 8:**
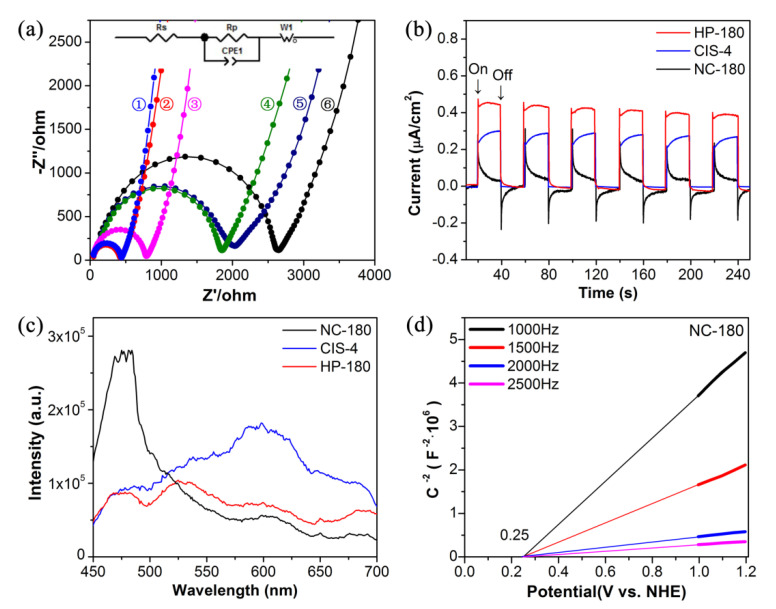
(**a**) Nyquist plots of ① HP-190, ② HP-180, ③ HP-150, ④ HP-120, ⑤ CIS-4, ⑥ NC-180; (**b**) transient photocurrent spectra of HP-180, CIS-4 and NC-180; (**c**) photoluminescence spectra of CIS-4, NC-180, HP-180; and (**d**) Mott-Schottky plots of NC-180.

To investigate the separation and transfer efficiency of photo-generated charge carriers in CdIn_2_S_4_/In(OH)_3_/NiCr-LDH, electrochemical impedance spectroscopy (EIS), transient photocurrent response and photoluminescence (PL) measurements were carried out. Figure S9 showed samples and as-prepared working electrode for EIS and transient photocurrent measurements. Figure 8a showed Nyquist plots of HP-120, HP-150, HP-180 and HP-190 together with CdIn_2_S_4_ and NiCr-LDH. It can be found that the semicircles of HP-120, HP-150, HP-180 and HP-190 have smaller diameters than that of the counterparts CdIn_2_S_4_ and NiCr-LDH. The semicircles in the EIS spectra can be ascribed to the contribution from the charge transfer resistance (R_p_) and the constant phase element (CPE1) at the photocatalyst/electrolyte interface, while the inclined line is associated with the ion diffusion in the electrolyte [60,61]. The corresponding equivalent circuit is shown as the inset of Figure 8a. The extracted R_p_ values are 391.8, 339.6, 683.7, 1665, 1686 and 2356 Ω for HP-190, HP-180, HP-150, HP-120, CdIn_2_S_4_ and NiCr-LDH, respectively. It can be found that samples HP-190, HP-180 and HP-150 have smaller R_p_ values than CdIn_2_S_4_ and NiCr-LDH, and HP-180 has the smallest R_p_ value, indicating enhanced electron transfer and suppressed charge recombination in HP-190, HP-180 and HP-150, which might be due to the multiple intimate-contact interface in the CdIn_2_S_4_/In(OH)_3_/NiCr-LDH samples. The lower impedance and higher charge transfer efficiency is beneficial for promoting photocatalytic performance, which is consistent with the results of photocatalytic H_2_ evolution. As illustrated in Figure 8b, CdIn_2_S_4_/In(OH)_3_/NiCr-LDH exhibited higher photocurrent density response than that of the counterparts CdIn_2_S_4_ and NiCr-LDH, indicating improved charge separation efficiency. Figure 8c showed the PL spectra of CdIn_2_S_4_, NiCr-LDH and HP-180. It is known that when a photocatalyst is exposed to light irradiation, electrons are excited from the valence band to the conduction band. After that, photo-generated electrons can recombine with holes to emit light (radiative recombination), trapped by deep energy levels associated with defects in bulk, and migrate to the surface to potentially participate in photocatalytic reactions. HP-180 had a lower emission intensity than CdIn_2_S_4_ and NiCr-LDH, implying reduced radiative recombination of electrons and holes in CdIn_2_S_4_/In(OH)_3_/NiCr-LDH, and possibly more photo-generated charge carriers to contribute to photocatalytic reactions. Therefore, the results of the EIS, photocurrent response and PL measurements demonstrated that the construction of CdIn_2_S_4_/In(OH)_3_/NiCr-LDH can accelerate the separation and migration efficiency of photo-generated electrons and holes, which are highly favorable for photocatalytic H_2_ evolution and Cr(VI) reduction.

Furthermore, the conduction band and valence band potentials of the component semiconductors are determined based on the Mott-Schottky plots and UV-Vis spectra analysis. The derived flat-band potentials of CdIn_2_S_4_, In(OH)_3_ and NiCr-LDH are around −1.13 eV [62], −0.58 eV [45], and 0.25 eV (Figure 8d) versus NHE, respectively. The CB potential of undoped n-type semiconductor is typically 0.3 eV more negative than the flat-band potentials [63], and thus the CB potentials of CdIn_2_S_4_, In(OH)_3_, and NiCr-LDH are around −1.43, −0.88 and −0.05 eV versus NHE, respectively. Subtracting the corresponding band gap energies of 2.06, 4.15 [45], and 2.46 eV, the VB potentials of CdIn_2_S_4_, In(OH)_3_, and NiCr-LDH are located at about 0.63, 3.27 and 2.41 eV (vs. NHE), respectively.

**Scheme 1:**
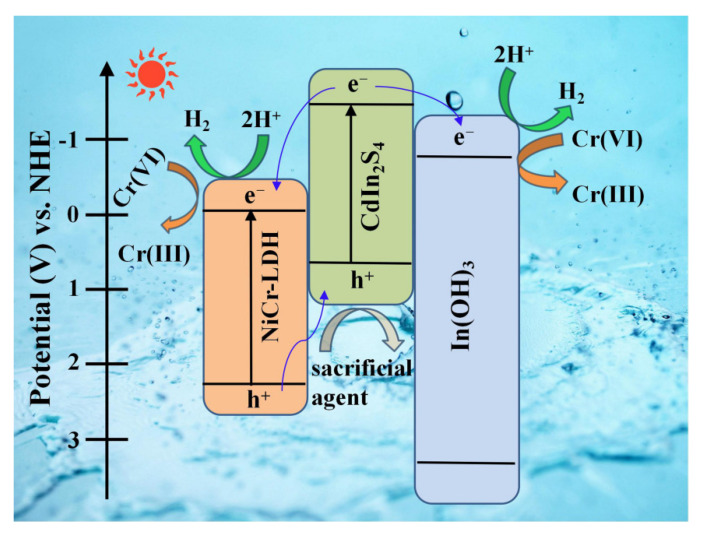
Illustration of the transfer process of the photo-excited charge carriers and plausible photocatalytic mechanism for H_2_ production and Cr(VI) reduction in the CdIn_2_S_4_/In(OH)_3_/NiCr-LDH heterostructure photocatalyst.

Based on the above results, a possible mechanism with facilitated charge transfer process and improved photocatalytic activity of CdIn_2_S_4_/In(OH)_3_/NiCr-LDH heterostructure photocatalysts in this work was proposed in Scheme 1. CdIn_2_S_4_ and NiCr-LDH with strong visible-light absorption can be easily excited under light illumination and engender electrons and holes in the CB and VB, respectively. As the CB of CdIn_2_S_4_ (−1.43 eV vs. NHE) is more negative than that of In(OH)_3_ (−0.88 eV vs. NHE) and NiCr-LDH (−0.05 eV vs. NHE), the photo-generated electrons in the CB of CdIn_2_S_4_ are transferred to the CB of In(OH)_3_ and NiCr-LDH, where the electrons can effectively reduce H^+^ to produce H_2_ molecules. Meanwhile, the holes accumulated in the VB of CdIn_2_S_4_ could be quenched by the sacrificial agent (S^2−^, SO_3_^2−^). During the photocatalytic reaction, the multi-interfaces with strong interactions play a vital role in the reaction as they ensure an unimpeded pathway for the fast electron transfer. In the photocatalytic reduction of Cr(VI) reaction, the photo-generated electrons can reduce Cr_2_O_7_^2−^ to Cr(III) (0.51 eV vs. NHE) [40]. Regarding the existence of the In_2_S_3_ impurity phase, it has been reported by Ma et al. that In_2_S_3_ also has strong visible light absorption and similar CB potential as that of CdIn_2_S_4_. Therefore we are not going to discuss charge transfer between CdIn_2_S_4_ and the impurity phase of In_2_S_3_ here [64].

## 4. Conclusions

In summary, CdIn_2_S_4_/In(OH)_3_/NiCr-LDH heterostructure photocatalysts were prepared by a simple and cost-effective hydrothermal method through one-pot depositing CdIn_2_S_4_ and In(OH)_3_ on NiCr-LDH. The obtained CdIn_2_S_4_/In(OH)_3_/NiCr-LDH exhibited improved photocatalytic performance on H_2_ production and Cr(VI) reduction. The optimal CdIn_2_S_4_/In(OH)_3_/NiCr-LDH sample HP-180 has a photocatalytic H_2_ evolution rate of 1093 μmol·g^−1^·h^−1^, which is 10.9 and 58.7 times that of the counterparts CIS-4 and NC-180, respectively. HP-180 showed good stability and the H_2_ evolution rate remained at 91% after four consecutive runs. Furthermore, HP-180 also showed enhanced photocatalytic reduction of Cr(VI), and the removal efficiency increased from 75% for CIS-4 and 6% for NC-180 to 97% after 120 min illumination. The photocatalytic mechanism was investigated by combining UV-Vis DRS, EIS, transient photocurrent response, PL and theoretical calculations. The improved photocatalytic activity is tentatively attributed to the boosted separation and transfer of photo-excited electrons and holes due to the multiple intimate-contact interfaces and strong visible-light absorption ability. This work can shed light on further design, and easy and cost-effective preparation of new LDH-based ternary nanocomposite photocatalysts for applications in energy conversion and environmental remediation.

## Data Availability

The data present in this study are available on request from the corresponding author.

## Supplementary Materials

The following are available online at https://www.mdpi.com/article/10.3390/nano11113122/s1, Figure S1: Light intensity versus wavelength of a 300 W Xe lamp (PLS-SXE 300) used in photocatalytic H2 evolution experiments, supplied from Beijing Perfect Light Technology, Co. Ltd., Figure S2: XRD patterns of (a) NC-120, NC-150, NC-180, NC-190, (b) CIS-4, CIS-4.4, CIS-6 and CIS-8, (c) HP-180, HP-180-1 and HP-180-2, and (d) HP-180, HP-180-3 and HP-180-4, Figure S3: (a) TEM and (b) HRTEM images of HP-180 recorded from a different area, other than that used in Figure 3c,d, Figure S4: XPS survey spectra of CIS-4, NC-180 and HP-180, revealing that the heterostructure catalyst is mainly com-posed of Cd, Cr, In, Ni, C, O and S elements, Figure S5: The band structure (a,b) and partial density of states (PDOS) (c,d) of CIS-4 and NC-180, respectively, Figure S6: Photocatalytic H2 production when 10, 50 and 100 mg HP-180 were used. It can be observed that the photocatalytic activity is related to the amount of catalyst and 50 mg leads to higher H_2_ yield, Figure S7: (a) Photocatalytic H_2_ production and (b) evolution rate over HP-180, HP-180-1 and HP-180-2. (c) Photocatalytic H_2_ production and (d) evolution rate over HP-180, HP-180-3 and HP-180-4, Figure S8: Photocatalytic reduction efficiencies Cr(VI) over HP-180 in the presence of electron scavenger (AgNO_3_, 1 mM), Figure S9: Photographs of powder samples and preparation of film samples for EIS and photocurrent response measure-ments. From left to right ① HP-190, ② HP-180, ③ HP-150, ④ HP-120, ⑤ CIS-4, ⑥NC-180.

## References

[1] Takata T., Jiang J.Z., Sakata Y., Nakabayashi M., Shibata N., Nandal V., Seki K., Hisatomi T., Domen K. (2020). Photocatalytic water splitting with a quantum efficiency of almost unity. Nature.

[2] Dawood F., Anda M., Shafiullah G.M. (2020). Hydrogen production for energy: An overview. Int. J. Hydrog. Energ..

[3] Wang Y., Diaz D.F.R., Chen K.S., Wang Z., Adroher X.C. (2020). Materials, technological status, and fundamentals of PEM fuel cells—A review. Mater. Today.

[4] Sarma G.K., Gupta S.S., Bhattacharyya K.G. (2019). Nanomaterials as versatile adsorbents for heavy metal ions in water: A review. Environ. Sci. Pollut. R..

[5] Zhang W.H., Mohamed A.R., Ong W.J. (2020). Z-Scheme photocatalytic systems for carbon dioxide reduction: Where are we now?. Angew. Chem. Int. Edit..

[6] Liang Z.Z., Shen R.C., Ng Y.H., Zhang P., Xiang Q.J., Li X. (2020). A review on 2D MoS_2_ cocatalysts in photocatalytic H_2_ production. J. Mater. Sci. Technol..

[7] Zhitkovich A. (2011). Chromium in Drinking Water: Sources, Metabolism, and Cancer Risks. Chem. Res. Toxicol..

[8] Zhao Z., An H., Lin J., Feng M., Murugadoss V., Ding T., Liu H., Shao Q., Mai X., Wang N. (2019). Progress on the Photocatalytic Reduction Removal of Chromium Contamination. Chem. Rec..

[9] Wang Q., Domen K. (2020). Particulate Photocatalysts for Light-Driven Water Splitting: Mechanisms, Challenges, and Design Strategies. Chem. Rev..

[10] Ong W.-J., Tan L.-L., Ng Y.H., Yong S.-T., Chai S.-P. (2016). Graphitic Carbon Nitride (g-C_3_N_4_)-Based Photocatalysts for Artificial Photosynthesis and Environmental Remediation: Are We a Step Closer to Achieving Sustainability?. Chem. Rev..

[11] Hunge Y.M., Yadav A.A., Mathe V.L. (2019). Photocatalytic hydrogen production using TiO_2_ nanogranules prepared by hydrothermal route. Chem. Phys. Lett..

[12] Ong W.-J., Shak K.P.Y. (2020). 2D/2D heterostructured photocatalysts: An emerging platform for artificial photosynthesis. Sol. RRL.

[13] Qin Y., Li H., Lu J., Feng Y., Meng F., Ma C., Yan Y., Meng M. (2020). Synergy between van der waals heterojunction and vacancy in ZnIn_2_S_4_/g-C_3_N_4_ 2D/2D photocatalysts for enhanced photocatalytic hydrogen evolution. Appl. Catal. B Environ..

[14] Xia P.F., Cao S.W., Zhu B.C., Liu M.J., Shi M.S., Yu J.G., Zhang Y.F. (2020). Designing a 0D/2D S-Scheme Heterojunction over Polymeric Carbon Nitride for Visible-Light Photocatalytic Inactivation of Bacteria. Angew. Chem. Int. Edit..

[15] Reddy C.V., Reddy K.R., Shetti N.P., Shim J., Aminabhavi T.M., Dionysiou D.D. (2020). Hetero-nanostructured metal oxide-based hybrid photocatalysts for enhanced photoelectrochemical water splitting—A review. Int. J. Hydrog. Energ..

[16] Ng B.J., Putri L.K., Kong X.Y., Teh Y.W., Pasbakhsh P., Chai S.P. (2020). Z-Scheme photocatalytic systems for solar water splitting. Adv. Sci..

[17] Xu Q.L., Zhang L.Y., Cheng B., Fan J.J., Yu J.G. (2020). S-Scheme heterojunction photocatalyst. Chem.

[18] Di T., Xu Q., Ho W., Tang H., Xiang Q., Yu J. (2019). Review on metal sulphide-based Z-scheme photocatalysts. Chemcatchem.

[19] Yadav A.A., Hunge Y.M., Kang S.-W. (2021). Spongy ball-like copper oxide nanostructure modified by reduced graphene oxide for enhanced photocatalytic hydrogen production. Mater. Res. Bull..

[20] Yadav A.A., Hunge Y.M., Kang S.-W. (2021). Porous nanoplate-like tungsten trioxide/reduced graphene oxide catalyst for sonocatalytic degradation and photocatalytic hydrogen production. Surf. Interf..

[21] Jiang Y., Chen H.-Y., Li J.-Y., Liao J.-F., Zhang H.-H., Wang X.-D., Kuang D.-B. (2020). Z-Scheme 2D/2D heterojunction of CsPbBr_3_/Bi_2_WO_6_ for improved photocatalytic CO_2_ reduction. Adv. Funct. Mater..

[22] Han Q., Li L., Gao W., Shen Y., Wang L., Zhang Y., Wang X., Shen Q., Xiong Y., Zhou Y. (2021). Elegant construction of ZnIn_2_S_4_/BiVO_4_ hierarchical heterostructures as direct Z-Scheme photocatalysts for efficient CO_2_ photoreduction. ACS Appl. Mater. Inter..

[23] Ng S.-F., Lau M.Y.L., Ong W.-J. (2021). Engineering layered double hydroxide–based photocatalysts toward artificial photosynthesis: State-of-the-art progress and prospects. Sol. RRL.

[24] Zhang S., Zhao Y., Shi R., Zhou C., Waterhouse G.I.N., Wu L.-Z., Tung C.-H., Zhang T. (2020). Efficient photocatalytic nitrogen fixation over Cu^δ+^-modified defective ZnAl-layered double hydroxide nanosheets. Adv. Energy Mater..

[25] Zhang G., Zhang X., Meng Y., Pan G., Ni Z., Xia S. (2020). Layered double hydroxides-based photocatalysts and visible-light driven photodegradation of organic pollutants: A review. Chem. Eng. J..

[26] Yang Z.-Z., Wei J.-J., Zeng G.-M., Zhang H.-Q., Tan X.-F., Ma C., Li X.-C., Li Z.-H., Zhang C. (2019). A review on strategies to LDH-based materials to improve adsorption capacity and photoreduction efficiency for CO_2_. Coordin. Chem. Rev..

[27] Sideris P.J., Nielsen U.G., Gan Z., Grey C.P. (2008). Mg/Al ordering in layered double hydroxides revealed by multinuclear NMR spectroscopy. Science.

[28] Tao J., Yu X., Liu Q., Liu G., Tang H. (2021). Internal electric field induced S–scheme heterojunction MoS_2_/CoAl LDH for enhanced photocatalytic hydrogen evolution. J. Colloid Interf. Sci..

[29] Li S., Wang L., Li Y., Zhang L., Wang A., Xiao N., Gao Y., Li N., Song W., Ge L. (2019). Novel photocatalyst incorporating Ni-Co layered double hydroxides with P-doped CdS for enhancing photocatalytic activity towards hydrogen evolution. Appl. Catal. B Environ..

[30] Nayak S., Swain G., Parida K. (2019). Enhanced photocatalytic activities of RhB degradation and H_2_ evolution from in situ formation of the electrostatic heterostructure MoS_2_/NiFe LDH nanocomposite through the Z-Scheme mechanism via p–n heterojunctions. ACS Appl. Mater. Inter..

[31] Wu Y., Gong Y., Liu J., Chen T., Liu Q., Zhu Y., Niu L., Li C., Liu X., Sun C.Q. (2020). Constructing NiFe-LDH wrapped Cu_2_O nanocube heterostructure photocatalysts for enhanced photocatalytic dye degradation and CO_2_ reduction via Z-scheme mechanism. J. Alloy. Compd..

[32] Li X., Yu Z., Shao L., Zeng H., Liu Y., Feng X. (2020). A novel strategy to construct a visible-light-driven Z-scheme (ZnAl-LDH with active phase/g-C_3_N_4_) heterojunction catalyst via polydopamine bridge (a similar “bridge” structure). J. Hazard. Mater..

[33] Wang J., Sun S., Zhou R., Li Y., He Z., Ding H., Chen D., Ao W. (2021). A review: Synthesis, modification and photocatalytic applications of ZnIn_2_S_4_. J. Mater. Sci. Technol..

[34] Li X., Sun Y., Xu J., Shao Y., Wu J., Xu X., Pan Y., Ju H., Zhu J., Xie Y. (2019). Selective visible-light-driven photocatalytic CO_2_ reduction to CH_4_ mediated by atomically thin CuIn_5_S_8_ layers. Nat. Energy.

[35] Zuo G., Wang Y., Teo W.L., Xie A., Guo Y., Dai Y., Zhou W., Jana D., Xian Q., Dong W. (2020). Ultrathin ZnIn_2_S_4_ nanosheets anchored on Ti_3_C_2_T_X_ MXene for photocatalytic H_2_ evolution. Angew. Chem. Int. Edit..

[36] Yadav A.A., Hunge Y.M., Kang S.-W. (2021). Ultrasound assisted synthesis of highly active nanoflower-like CoMoS_4_ electrocatalyst for oxygen and hydrogen evolution reactions. Ultrason. Sonochem..

[37] Zhuge Z., Liu X., Chen T., Gong Y., Li C., Niu L., Xu S., Xu X., Alothman Z.A., Sun C.Q. (2021). Highly efficient photocatalytic degradation of different hazardous contaminants by CaIn_2_S_4_-Ti_3_C_2_T_x_ Schottky heterojunction: An experimental and mechanism study. Chem. Eng. J..

[38] Gao B., Dong S., Liu J., Liu L., Feng Q., Tan N., Liu T., Bo L., Wang L. (2016). Identification of intermediates and transformation pathways derived from photocatalytic degradation of five antibiotics on ZnIn_2_S_4_. Chem. Eng. J..

[39] Pei C.-Y., Chen Y.-G., Wang L., Chen W., Huang G.-B. (2021). Step-scheme WO_3_/CdIn_2_S_4_ hybrid system with high visible light activity for tetracycline hydrochloride photodegradation. Appl. Surf. Sci..

[40] Cheng C., Chen D., Li N., Li H., Xu Q., He J., Lu J. (2021). Construction of hollow In2S3/CdIn2S4 heterostructures with high efficiency for Cr(vi) reduction. Environ. Sci. Nano.

[41] Chen W., Chang L., Ren S.-B., He Z.-C., Huang G.-B., Liu X.-H. (2020). Direct Z-scheme 1D/2D WO2.72/ZnIn2S4 hybrid photocatalysts with highly-efficient visible-light-driven photodegradation towards tetracycline hydrochloride removal. J. Hazard. Mater..

[42] Guo J., Wang L., Wei X., Alothman Z.A., Albaqami M.D., Malgras V., Yamauchi Y., Kang Y., Wang M., Guan W. (2021). Direct Z-scheme CuInS_2_/Bi_2_MoO_6_ heterostructure for enhanced photocatalytic degradation of tetracycline under visible light. J. Hazard. Mater..

[43] Zhang G., Chen D., Li N., Xu Q., Li H., He J., Lu J. (2020). Construction of Hierarchical Hollow Co_9_S_8_/ZnIn_2_S_4_ Tubular Heterostructures for Highly Efficient Solar Energy Conversion and Environmental Remediation. Angew. Chem. Int. Edit..

[44] Li Y., Hou Y., Fu Q., Peng S., Hu Y.H. (2017). Oriented growth of ZnIn2S4/In(OH)3 heterojunction by a facile hydrothermal transformation for efficient photocatalytic H2 production. Appl. Catal. B Environ..

[45] Geng M., Peng Y., Zhang Y., Guo X., Yu F., Yang X., Xie G., Dong W., Liu C., Li J. (2019). Hierarchical ZnIn_2_S_4_: A promising cocatalyst to boost visible-light-driven photocatalytic hydrogen evolution of In(OH)_3_. Int. J. Hydrog. Energ..

[46] Zhao H., Zhang T., Shan D., Zhu Y., Gao G., Liu Y., Liu J., Liu M., You W. (2020). ZnIn_2_S_4_/In(OH)_3_ hollow microspheres fabricated by one-step l-cysteine-mediated hydrothermal growth for enhanced hydrogen production and MB degradation. Int. J. Hydrog. Energ..

[47] Ye L., Wen Z., Li Z., Huang H. (2020). Hierarchical Architectured Ternary Nanostructures Photocatalysts with In(OH)_3_ Nanocube on ZnIn_2_S_4_/NiS Nanosheets for Photocatalytic Hydrogen Evolution. Solar RRL.

[48] Zhao J., Yan X., Zhao N., Li X., Lu B., Zhang X., Yu H. (2018). Cocatalyst designing: A binary noble-metal-free cocatalyst system consisting of ZnIn_2_S_4_ and In(OH)_3_ for efficient visible-light photocatalytic water splitting. RSC Adv..

[49] Li X., Yan X., Zhao N., Zhao J., Lu B., Zhang X., Zhang X., Yu H. (2018). Facile synthesis of ternary CdIn_2_S_4_/In(OH)_3_/Zn_2_GeO_4_ nanocomposite with enhanced visible-light photocatalytic H_2_ evolution. J. Photoch. Photobio. A.

[50] Tan Y.-X., Chai Z.-M., Wang B.-H., Tian S., Deng X.-X., Bai Z.-J., Chen L., Shen S., Guo J.-K., Cai M.-Q. (2021). Boosted photocatalytic oxidation of toluene into benzaldehyde on CdIn_2_S_4_-CdS: Synergetic effect of compact heterojunction and S-vacancy. ACS Catal..

[51] Zhao Y., Zhang S., Li B., Yan H., He S., Tian L., Shi W., Ma J., Wei M., Evans D.G. (2011). A family of visible-light responsive photocatalysts obtained by dispersing CrO_6_ octahedra into a hydrotalcite matrix. Chem. Eur. J..

[52] Wang H., Xia Y., Li H., Wang X., Yu Y., Jiao X., Chen D. (2020). Highly active deficient ternary sulfide photoanode for photoelectrochemical water splitting. Nat. Commun..

[53] Hu J., Chen C., Zheng Y., Zhang G., Guo C., Li C.M. (2020). Spatially separating redox centers on Z-Scheme ZnIn_2_S_4_/BiVO_4_ hierarchical heterostructure for highly efficient photocatalytic hydrogen evolution. Small.

[54] Tonda S., Kumar S., Bhardwaj M., Yadav P., Ogale S. (2018). g-C_3_N_4_/NiAl-LDH 2D/2D hybrid heterojunction for high-performance photocatalytic reduction of CO_2_ into renewable fuels. ACS Appl. Mater. Inter..

[55] Sahoo D.P., Nayak S., Reddy K.H., Martha S., Parida K. (2018). Fabrication of a Co(OH)_2_/ZnCr LDH “p–n” heterojunction photocatalyst with enhanced separation of charge carriers for efficient visible-light-driven H_2_ and O_2_ evolution. Inorg. Chem..

[56] Rodriguez-Rivas F., Pastor A., de Miguel G., Cruz-Yusta M., Pavlovic I., Sánchez L. (2020). Cr^3+^ substituted Zn-Al layered double hydroxides as UV–Vis light photocatalysts for NO gas removal from the urban environment. Sci. Total. Environ..

[57] Gao F., Lei R., Huang X., Yuan J., Jiang C., Feng W., Zhang L., Liu P. (2021). In situ etching growth of defective ZnS nanosheets anchored vertically on layered-double-hydroxide microflowers for accelerated photocatalytic activity. Appl. Catal. B Environ..

[58] Meng F., Qin Y., Lu J., Lin X., Meng M., Sun G., Yan Y. (2021). Biomimetic design and synthesis of visible-light-driven g-C_3_N_4_ nanotube @polydopamine/NiCo-layered double hydroxides composite photocatalysts for improved photocatalytic hydrogen evolution activity. J. Colloid Interf. Sci..

[59] He J.-Y., Zhang D., Wang X.-J., Zhao J., Li Y.-P., Liu Y., Li F.-T. (2021). Phosphorylation of NiAl-layered double hydroxide nanosheets as a novel cocatalyst for photocatalytic hydrogen evolution. Int. J. Hydrog. Energ..

[60] Zhao Y., Zhao Y., Waterhouse G.I.N., Zheng L., Cao X., Teng F., Wu L.-Z., Tung C.-H., O’Hare D., Zhang T. (2017). Layered-Double-Hydroxide Nanosheets as Efficient Visible-Light-Driven Photocatalysts for Dinitrogen Fixation. Adv. Mater..

[61] Liu H., Guo H., Liu B., Liang M., Lv Z., Adair K.R., Sun X. (2018). Few-Layer MoSe2 Nanosheets with Expanded (002) Planes Confined in Hollow Carbon Nanospheres for Ultrahigh-Performance Na-Ion Batteries. Adv. Funct. Mater..

[62] Wang S., Guan B.Y., Lu Y., Lou X.W.D. (2017). Formation of Hierarchical In_2_S_3_–CdIn_2_S_4_ Heterostructured Nanotubes for Efficient and Stable Visible Light CO_2_ Reduction. J. Am. Chem. Soc..

[63] Xiaofei Y., Lin T., Xiaolong Z., Hua T., Qinqin L., Guisheng L. (2019). Interfacial optimization of g-C_3_N_4_-base Z-scheme heterojunction toward synergistic enhancement of solar-driven photocatalytic oxygen evolution. Appl. Catal. B Environ..

[64] Ma D., Shi J.-W., Zou Y., Fan Z., Shi J., Cheng L., Sun D., Wang Z., Niu C. (2018). Multiple carrier-transfer pathways in a flower-like In_2_S_3_/CdIn_2_S_4_/In_2_O_3_ ternary heterostructure for enhanced photocatalytic hydrogen production. Nanoscale.

