# Supplementary material for: CdIn2S4/In(OH)3/NiCr-LDH Multi-Interface Heterostructure Photocatalyst for Enhanced Photocatalytic H2 Evolution and Cr(VI) Reduction
Source: Nanomaterials (Basel). 2021 Nov 19;11(11):3122. doi: 10.3390/nano11113122 (PMC8619374; doi:10.3390/nano11113122)
Supplement: Supplementary file 1 [file nanomaterials-11-03122-s001.zip › nanomaterials-1406583-supplementary.pdf]

nanomaterials

Supporttting Information

$CdIn_2S_4/In(OH)_3$/NiCr-LDH Multi-Interface Heterostructure Photocatalyst for Enhanced Photocatalytic H_2 Evolution and Cr(VI) Reduction

Rao Fu [1,2], Yinyan Gong [2,*], Can Li [2], Lengyuan Niu [2] and Xinjuan Liu [2]

1 College of Materials and Chemistry, China Jiliang University, Hangzhou 310018, China; furao0802@163.com
2 Institute of Optoelectronic Materials and Devices, College of Optical and Electronic Technology, China Jiliang University, Hangzhou 310018, China; canli1983@gmail.com (C.L.); niulengyuan@163.com (L.N.); lxj669635@126.com (X.L.)
* Correspondence: 13a0502075@cjlu.edu.cn; Tel./Fax: +86-571-8687-2363

1. Characterization of as-prepared photocatalysts

Field-emission scanning electron microscopy (SEM, Hitachi S-4800) and transmission electron microscopy (TEM, Tecnai G2 F20) were carried out to investigate the morphology and microstructure of as-prepared samples. X-ray power diffraction (XRD) measurements were performed on Bruker D2 diffractometer using Cu Kα irradiation (λ = 0.154 nm). The surface elements and chemical states were analyzed by X-ray photoelectron spectroscopy (XPS, ESCALAB 250Xi) using Al Kα irradiation (1486.6 eV). The UV-Vis diffuse reflectance spectra (DRS) were measured using a Perkin Elmer UV-Vis-NIR spectrometer Lamda 750 equipped with an integrating sphere attachment.

The photoelectrochemical properties of samples were tested on a CHI 660E electrochemical workstation in a three-electrode configuration. To prepare working electrodes, photocatalysts and 5 wt% cellulose binder were homogeneously mixed in terpineol, coated on ITO slides (active area 1 cm^2) and dried at 60 °C overnight. Pt foil and Ag/AgCl were used as counter and reference electrodes, respectively. The electrochemical impedance spectroscopy (EIS) measurements were measured using 50 ppm Cr(VI) solution as electrolyte. For transient photocurrent measurements, a 300 W Xe lamp equipped with a chopper was used as light source and 0.5 M Na_2SO_4 as electrolyte.

2. Computational method and model

The first principles calculations in the framework of DFT were carried out based on the Cambridge Sequential Total Energy Package known as CASTEP [1]. The exchange-correlation functional under the generalized gradient approximation (GGA) [2] with norm-conserving pseudopotentials and Perdew–Burke–Ernzerhof (PBE) functional was adopted to describe the electron–electron interaction [3]. An energy cutoff of 750 eV was used and a k-point sampling set of 11 × 11 × 11 were tested to be converged. A force tolerance of 0.01 eV Å^{-1}, energy tolerance of 5.0×10^{-7}eV per atom and maximum displacement of 5.0×10^{-4} Å were considered. The $CdIn_2S_4$ and NiCr-LDH bulk were built.

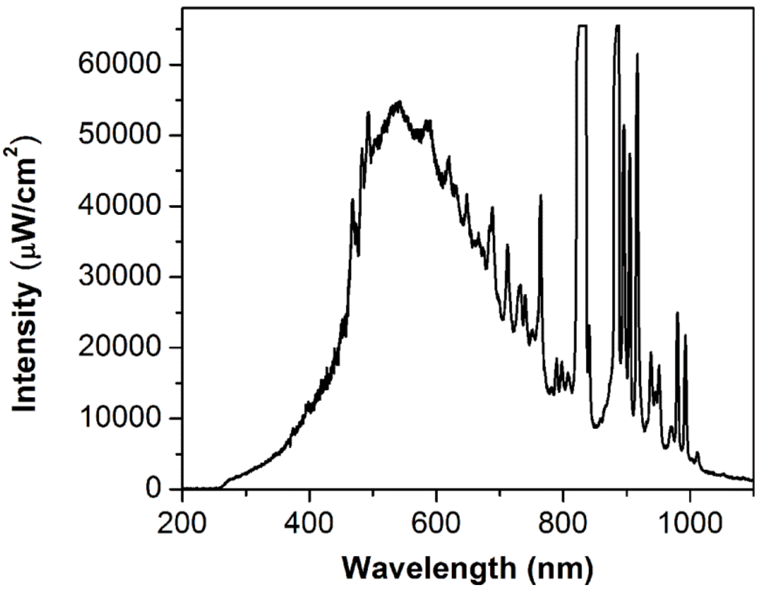

Figure S1. Light intensity versus wavelength of a 300 W Xe lamp (PLS-SXE 300) used in photocatalytic H_2 evolution experiments, supplied from Beijing Perfect Light Technology, Co. Ltd.

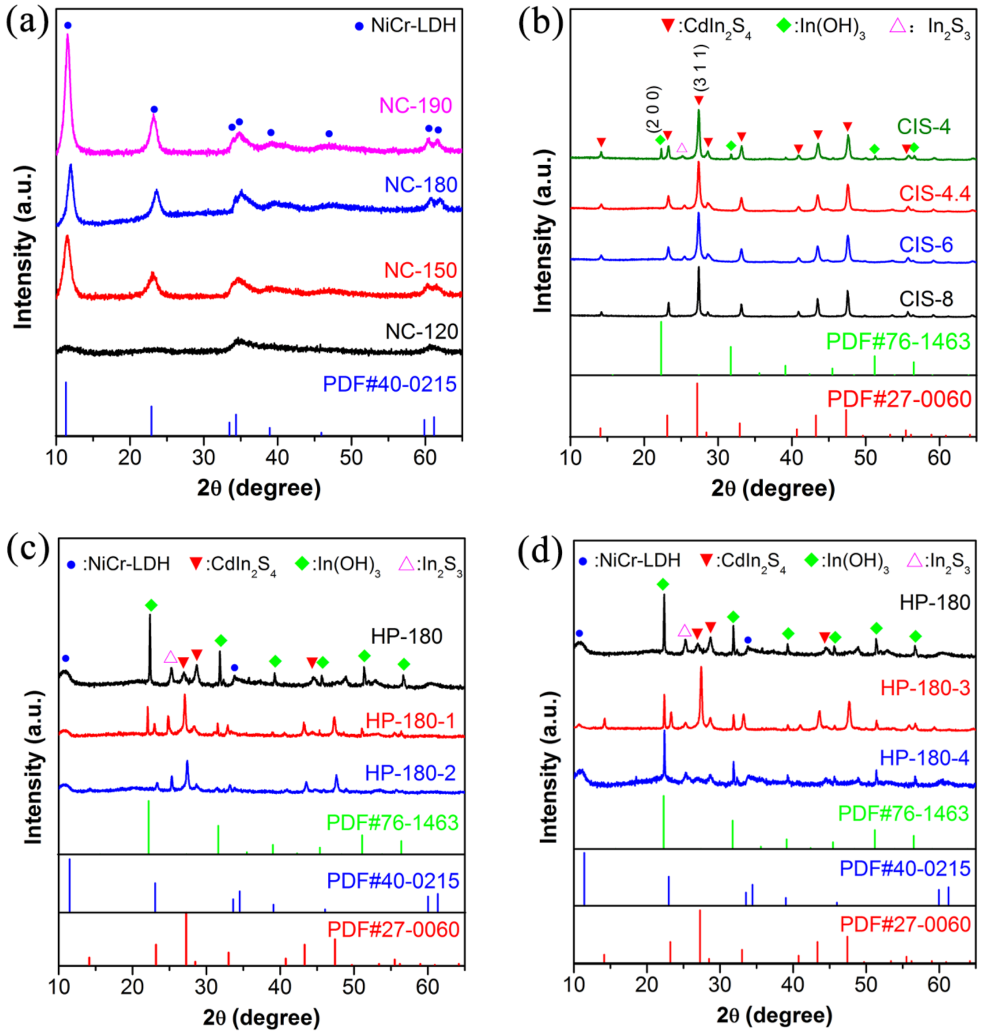

Figure S2. XRD patterns of (**a**) NC-120, NC-150, NC-180, NC-190, (**b**) CIS-4, CIS-4.4, CIS-6 and CIS-8, (**c**) HP-180, HP-180-1 and HP-180-2, and (**d**) HP-180, HP-180-3 and HP-180-4.

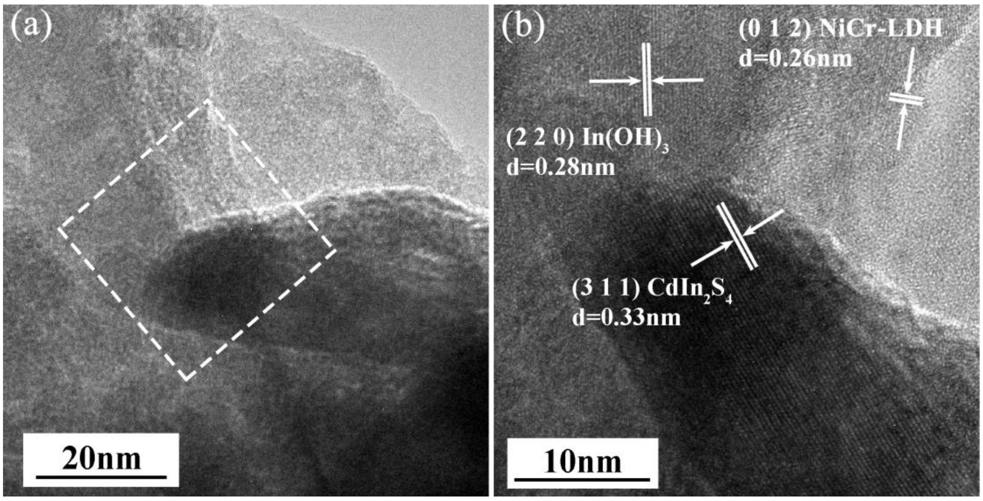

Figure S3. (**a**) TEM and (**b**) HRTEM images of HP-180 recorded from a different area, other than that used in Figures 3**c–d**.

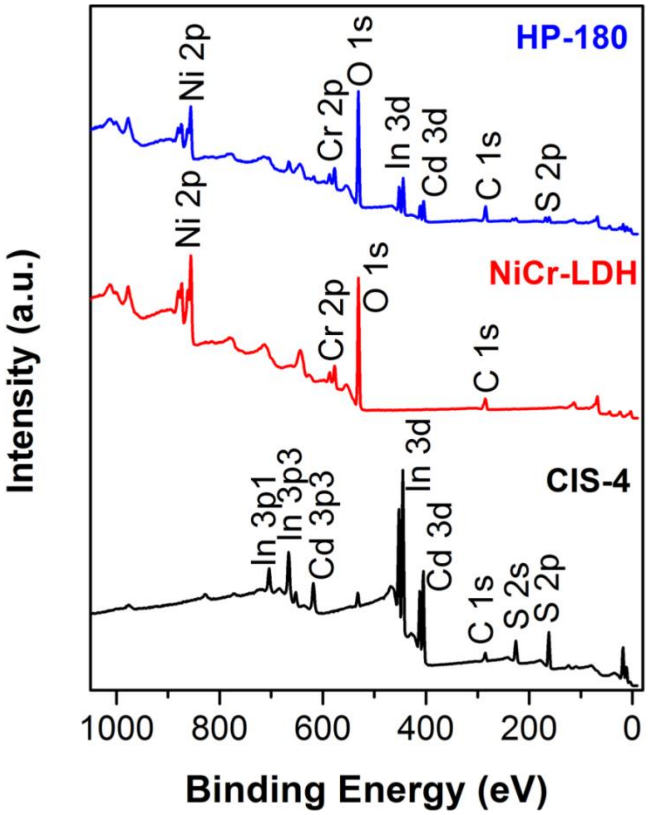

Figure S4. XPS survey spectra of CIS-4, NC-180 and HP-180, revealing that the heterostructure catalyst is mainly composed of Cd, Cr, In, Ni, C, O and S elements.

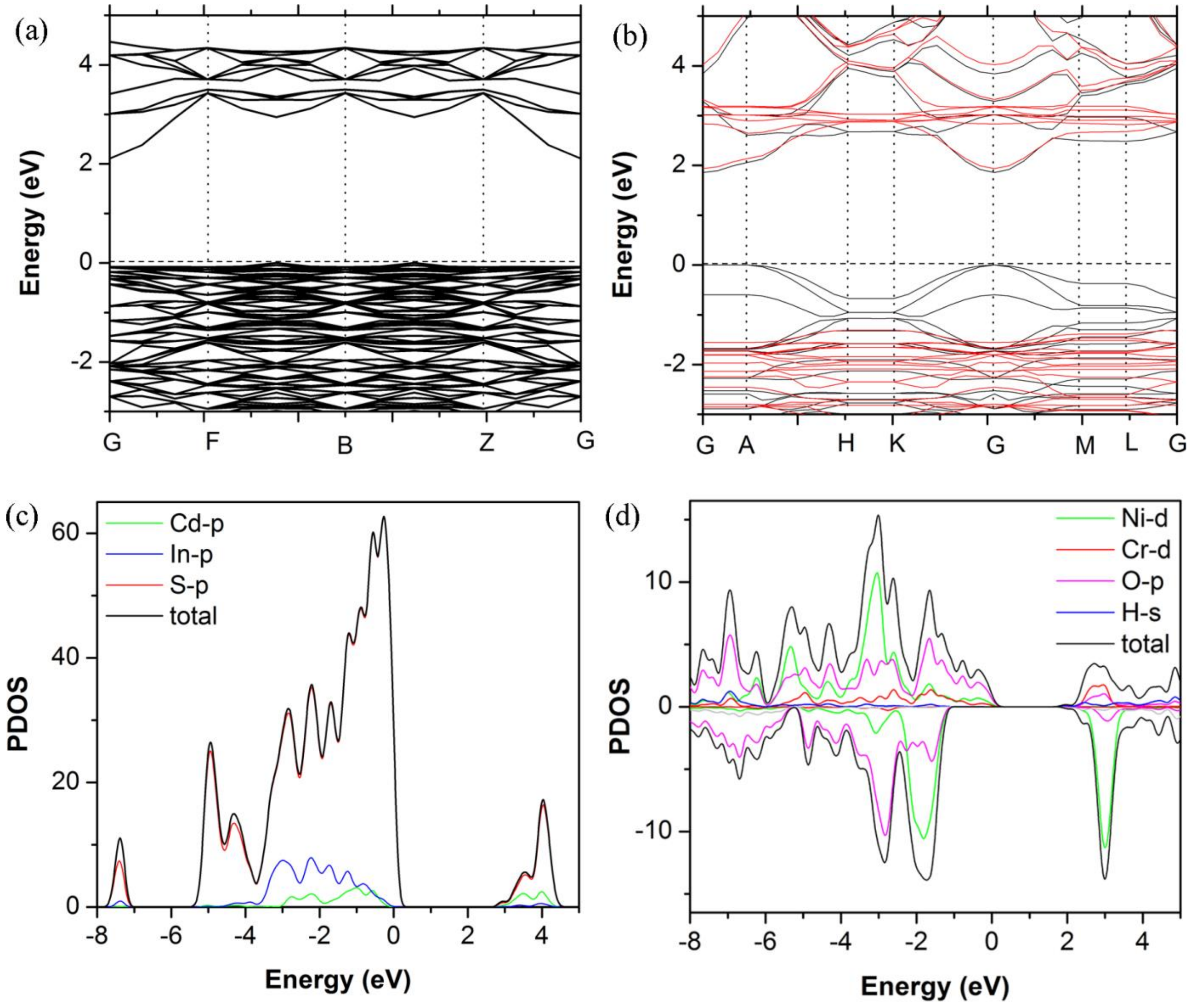

Figure S5. The band structure (**a, b**) and partial density of states (PDOS) (**c, d**) of CIS-4 and NC-180, respectively.

3. Quantum efficiency calculation

The number of incident photons (N) is 1.08 × 10^{21} as calculated by Equation (S1). The amount of H_2 molecules generated over HP-180 in 4 hours was 14.93 μmol. The AQY was calculated using Equation (S2).

$$N = \frac{E\lambda}{hc} = \frac{5.01\times10^{-3}\times7.1\times4\times3600\times420\times10^{-9}}{6.626\times10^{-34}\times3\times10^{8}}$$

Copyright: © 2021 by the authors. Licensee MDPI, Basel, Switzerland. This article is an open access article distributed under the terms and conditions of the Creative Commons Attribution (CC BY) license (https://creativecommons.org/licenses/by/4.0/).

$$= 1.08 \times 10^{21} \quad (S1)$$

$$AQY(\%) = \frac{2 \times \text{number of evolved } H_2 \text{ molecules}}{\text{number of incident photons}} \times 100\%$$

$$= \frac{2 \times 14.93 \times 10^{-6} \times 6.02 \times 10^{23}}{1.08 \times 10^{21}}$$

$$= 1.7\% \quad \text{(S2)}$$

Table S1. Summary of LDHs-based heterostructures for photocatalytic H_2 production.

**Photocatalyst**	**Reaction medium**	**Light source**	**H_2 production rate (μmol g^{-1} h^{-1})**	**Ref.**
$CdIn_2S_4/In(OH)_3$/ NiCr-LDH	$Na_2SO_3+Na_2S$	300W Xe λ ≥ 300nm	1093 AQY = 1.7% (λ = 420nm)	This work
CdS/ MgAl-LDH	CH_3OH	UV lamp	656	[4]
CdS/ NiV-LDH	$Na_2SO_3+Na_2S$	5W White LED	479.3	[5]
CdZnS/ ZnCr-LDH	CH_3OH	300W Xe 420-760nm	916.2 AQY=4.1% (420-760nm)	[6]
g-C_3N_4/ CoAl-LDH	TEOA	300W Xe AM 1.5	680.1	[7]
g-C_3N_4@pDA/ NiCo-LDH	TEOA	300W Xe λ ≥ 400nm	1555.1 AQY=4.5% (λ = 420nm)	[8]
g-C_3N_4/ ZnTi-LDH	CH_3OH	300W Xe λ ≥ 420nm	161.9	[9]
2D-rGO/ LTO/NiFe-LDH	TEOA	300W Xe λ ≥ 420nm	532	[10]
MoS_2/ CoAl-LDH	CH_3OH	300W Xe	17.1	[11]
P- NiAl-LDH/ g-C_3N_4	TEOA	300W Xe λ ≥ 400nm	1678.6 AQY=4.7% (λ = 420nm)	[12]
NiS/MgAl-LDH	CH_3OH	300W Xe λ ≥ 420nm	895	[13]
$NiTiO_3$/ CoAl-LDH	Ethylene glycol	300W Xe λ ≥ 420nm	594	[14]
ZnS/ ZnIn-LDH	$Na_2SO_3+Na_2S$	300W Xe λ ≥ 400nm	49.3 AQY=1.3% (λ = 415nm)	[15]

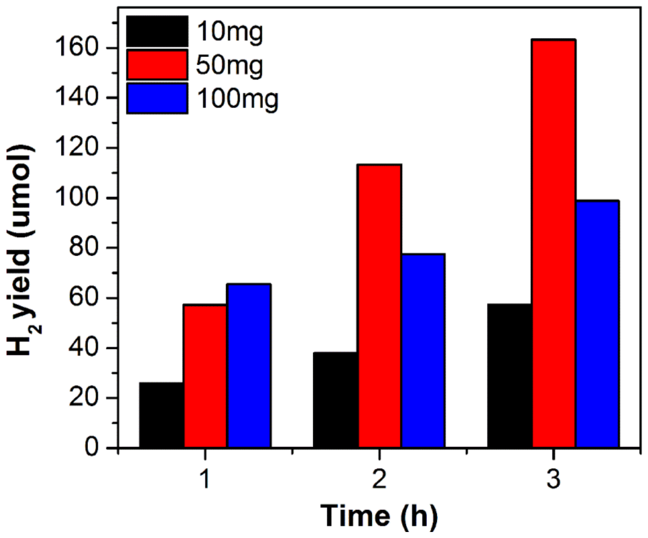

Figure S6 Photocatalytic H_2 production when 10, 50 and 100 mg HP-180 were used. It can be observed that the photocatalytic activity is related to the amount of catalyst and 50 mg leads to higher H_2 yield.

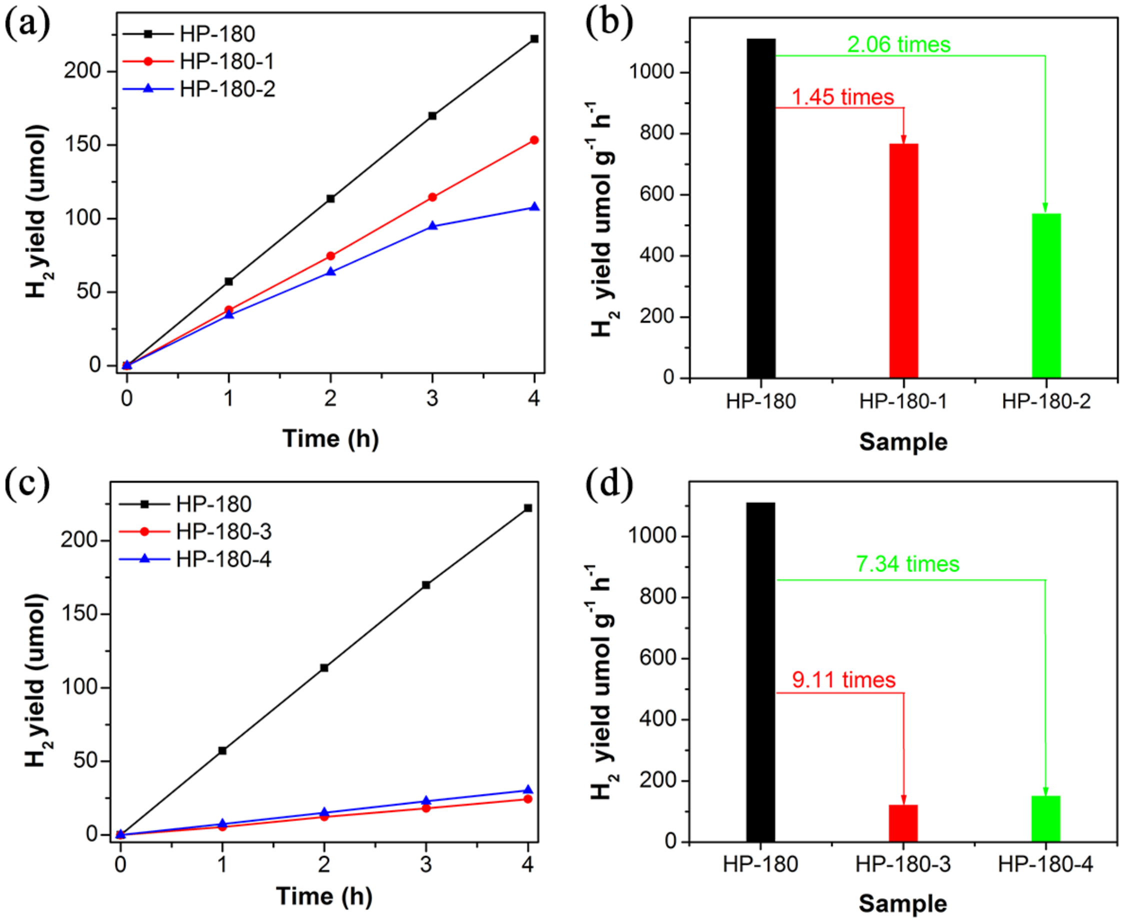

Figure S7. (**a**) Photocatalytic H_2 production and (**b**) evolution rate over HP-180, HP-180-1 and HP-180-2. (**c**) Photocatalytic H_2 production and (**d**) evolution rate over HP-180, HP-180-3 and HP-180-4.

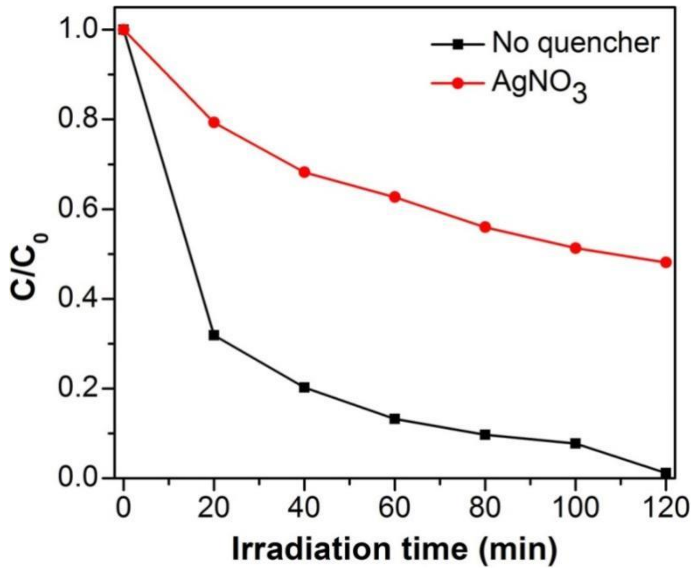

Figure S8. Photocatalytic reduction efficiencies Cr(VI) over HP-180 in the presence of electron scavenger ($AgNO_3$, 1mM).

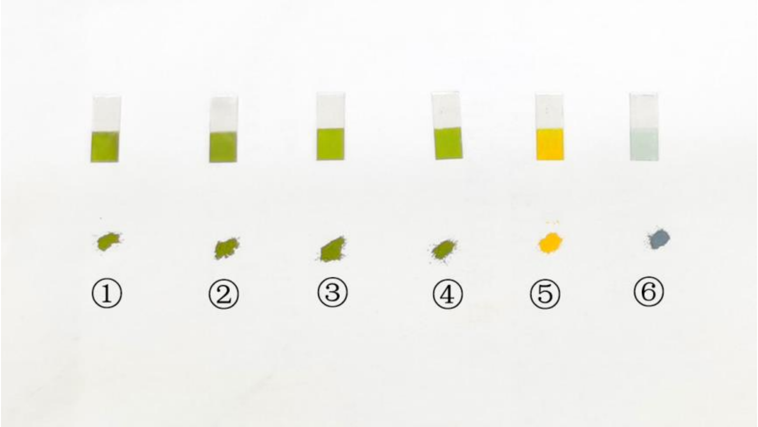

Figure S9. Photographs of powder samples and preparation of film samples for EIS and photocurrent response measurements. From left to right ① HP-190, ② HP-180, ③ HP-150, ④ HP-120, ⑤ CIS-4, ⑥NC-180.

References

1. Segall, M.D.; P.J.D. Lindan; M.J. Probert; C.J. Pickard; P.J. Hasnip; S.J. Clark, and M.C. Payne, First-principles simulation: ideas, illustrations and the CASTEP code. *J. Phys. condens. Mat.* **2002**, *14*, 2717–2744.
2. Perdew, J.P.; K. Burke, and M. Ernzerhof, Generalized Gradient Approximation Made Simple. *Phys. Rev. Lett.* **1996**, *77*, 3865–3868.
3. Hamann, D.R.; M. Schlüter, and C. Chiang, Norm-Conserving Pseudopotentials. *Phys. Rev. Lett.* **1979**, *43*, 1494–1497.
4. Mancipe, S.; F. Tzompantzi, and R. Gómez, Synthesis of CdS/MgAl layered double hydroxides for hydrogen production from methanol-water decomposition. *Appl. Clay Sci.* **2017**, *136*, 67–74.
5. Yang, M.; K. Wang; Y. Li; K. Yang, and Z. Jin, Pristine hexagonal CdS assembled with NiV LDH nanosheet formed p-n heterojunction for efficient photocatalytic hydrogen evolution. *Appl. Surf. Sci.* **2021**, *548*, 149212.
6. Yao, L.; D. Wei; D. Yan, and C. Hu, ZnCr Layered Double Hydroxide (LDH) Nanosheets Assisted Formation of Hierarchical Flower-Like CdZnS@LDH Microstructures with Improved Visible-Light-Driven H_2 Production. *Chem. Asian J.* **2015**, *10*, 630–636.
7. Zhang, J.; Q. Zhu; L. Wang; M. Nasir; S.-H. Cho, and J. Zhang, g-C_3N_4/CoAl-LDH 2D/2D hybrid heterojunction for boosting photocatalytic hydrogen evolution. *Int. J. Hydrogen Energ.* **2020**, *45*, 21331–21340.
8. Meng, F.; Y. Qin; J. Lu; X. Lin; M. Meng; G. Sun, and Y. Yan, Biomimetic design and synthesis of visible-light-driven g-C_3N_4 nanotube @polydopamine/NiCo-layered double hydroxides composite photocatalysts for improved photocatalytic hydrogen evolution activity. *J. Colloid Interf. Sci.* **2021**, *584*, 464–473.
9. Sun, D.; D. Chi; Z. Yang; Z. Xing; J. Yin; Z. Li; Q. Zhu, and W. Zhou, Mesoporous g-C_3N_4/Zn–Ti LDH laminated van der Waals heterojunction nanosheets as remarkable visible-light-driven photocatalysts. *Int. J. Hydrogen Energ.* **2019**, *44*, 16348–16358.

10. Boppella, R.; C.H. Choi; J. Moon, and D. Ha Kim, Spatial charge separation on strongly coupled 2D-hybrid of rGO/$La_2Ti_2O_7$/NiFe-LDH heterostructures for highly efficient noble metal free photocatalytic hydrogen generation. *Appl. Catal. B Environ.* **2018**, *239*, 178–186.
11. Tao, J.; X. Yu; Q. Liu; G. Liu, and H. Tang, Internal electric field induced S–scheme heterojunction MoS_2/CoAl LDH for enhanced photocatalytic hydrogen evolution. *J. Colloid. Interf. Sci.* **2021**, *585*, 470–479.
12. He, J.-Y.; D. Zhang; X.-J. Wang; J. Zhao; Y.-P. Li; Y. Liu, and F.-T. Li, Phosphorylation of NiAl-layered double hydroxide nanosheets as a novel cocatalyst for photocatalytic hydrogen evolution. *Int. J. Hydrogen Energ.* **2021**, *46*, 18977–18987.
13. Chen, J.; C. Wang; Y. Zhang; Z. Guo; Y. Luo, and C.-J. Mao, Engineering ultrafine NiS cocatalysts as active sites to boost photocatalytic hydrogen production of MgAl layered double hydroxide. *Appl. Surf. Sci.* **2020**, *506*, 144999.
14. Li, Y.B.; G.R. Wang; Y.B. Wang, and Z.L. Jin, Phosphating 2D CoAl LDH anchored on 3D self-assembled $NiTiO_3$ hollow rods for efficient hydrogen evolution. *Catal. Sci. Technol.* **2020**, *10*, 2931–2947.
15. Gao, F.; R. Lei; X. Huang; J. Yuan; C. Jiang; W. Feng; L. Zhang, and P. Liu, In situ etching growth of defective ZnS nanosheets anchored vertically on layered-double-hydroxide microflowers for accelerated photocatalytic activity. *Appl. Catal. B Environ.* **2021**, *292*, 120187.
